# XIAP is not required for human tumor cell survival in the absence of an exogenous death signal

**DOI:** 10.1186/1471-2407-10-11

**Published:** 2010-01-12

**Authors:** John Sensintaffar, Fiona L Scott, Robert Peach, Jeffrey H Hager

**Affiliations:** 1 Biology, Apoptos Inc, 10835 Road to the Cure, San Diego, CA 92130, USA; 2 Aragon Pharmaceuticals, 4215 Sorrento Valley Blvd., Suite 215 San Diego CA 92121, USA

## Abstract

**Background:**

The X-linked Inhibitor of Apoptosis (XIAP) has attracted much attention as a cancer drug target. It is the only member of the IAP family that can directly inhibit caspase activity *in vitro*, and it can regulate apoptosis and other biological processes through its C-terminal E3 ubiquitin ligase RING domain. However, there is controversy regarding XIAP's role in regulating tumor cell proliferation and survival under normal growth conditions *in vitro*.

**Methods:**

We utilized siRNA to systematically knock down XIAP in ten human tumor cell lines and then monitored both XIAP protein levels and cell viability over time. To examine the role of XIAP in the intrinsic versus extrinsic cell death pathways, we compared the viability of XIAP depleted cells treated either with a variety of mechanistically distinct, intrinsic pathway inducing agents, or the canonical inducer of the extrinsic pathway, TNF-related apoptosis-inducing ligand (TRAIL).

**Results:**

XIAP knockdown had no effect on the viability of six cell lines, whereas the effect in the other four was modest and transient. XIAP knockdown only sensitized tumor cells to TRAIL and not the mitochondrial pathway inducing agents.

**Conclusions:**

These data indicate that XIAP has a more central role in regulating death receptor mediated apoptosis than it does the intrinsic pathway mediated cell death.

## Background

An underlying feature of all human cancer is uncontrolled cell proliferation. However, for a tumor to increase in cell mass and malignant potential, the increase in replication rate must be accompanied by suppression of apoptosis [1]. While tumor cells can subvert many apoptotic regulators, the anti-apoptotic IAP family is thought to have a central role in this process.

There are eight IAPs in humans. All IAPs contain multiple functional domains that potentially modulate many biological processes, including apoptosis. For instance, IAPs have a role in cell-cycle regulation through mitotic spindle formation, ubiquitination of target proteins, and modulation of several signal transduction pathways [2]. Elevated IAP protein levels are common in many tumor types, and a wealth of data supports their role in suppressing cell death, although the exact mechanisms by which different IAPs mediate this effect remains unclear [3,4]

XIAP is the most thoroughly characterized of this family, and is the only member that can directly inhibit the proteolytic activity of caspases *in vitro *(reviewed in Eckelmen [5]). Caspase inhibition is mediated through an 80 amino acid motif, the Baculovirus IAP Repeat domain (BIR), common to all IAPs. By contrast, cIAPs can also directly interact with caspases, but largely to target caspase degradation through the ubiquitin ligase activity of the C-terminal RING domain [6]. Importantly, XIAP inhibits caspases at both the initiation phase (caspase-9) and the execution phase (caspases-3 and 7) of apoptosis [7]. In light of these activities, XIAP inhibition through small molecules or antisense has received considerable pharmaceutical industry focus, and multiple agents have progressed to clinical trials [3].

A hallmark of apoptotic cell death is the presence of proteolytically cleaved, catalytically active caspases. Viable cells of many well-studied cancer cell lines have been reported to exhibit high steady-state levels of activated caspases in the absence of other markers of cell death [8]. The resistance of these cells to apoptosis is thought to be mediated, at least in part, by XIAP. If XIAP function is essential for survival of these cancer cells, then its inhibition by pharmacological or genetic targeting should increase the rate of apoptosis, without the requirement of additional exogenous signals. XIAP loss of function has been studied extensively using various genetic tools including germ line deletion [9], somatic cell deletion [10], and both transient and stable mRNA knockdown. The results have varied widely; in some reports XIAP knockdown alone resulted in decreased viability, while other studies demonstrated no effect. Mice harboring XIAP null alleles are viable and do not exhibit any overt defects in developmental or homeostatic apoptosis, aside from a slight delay in mammary alveolar development [11,12]. These same XIAP null mice crossed to the TRAMP mouse model of prostate cancer did not result in an alteration in tumor progression, suggesting that XIAP is not a critical regulator of tumor apoptosis in this context [13]. However, loss of XIAP function can sensitize some cell lines *in vitro *to apoptosis mediated by activation of either the extrinsic, caspase 8 dependent pathway, using exogenous ligands such as TRAIL [10,14] and TNFα [15], or chemotherapeutic agents, which largely activate the intrinsic, caspase 9-dependent pathway [16-18]

Some of the different outcomes in XIAP depleted cells may be attributable to varying functional dependence on XIAP. On the other hand, there are conflicting reports even in the same cell line. In MCF-7 cells, Hu et. al., [19] reported that siRNA-mediated knockdown of XIAP had no effect on cell viability in the absence of an exogenous apoptotic stimulus. By contrast, Zhang et. al. [20] reported a 70% decrease in MCF-7 viability within 60 hr after transient siRNA-mediated loss of XIAP. Also, Lima et. al. [21] reported an approximately 50% decrease in viability in MCF-7 cells, 96 hr post transfection with XIAP-targeted siRNA. In another example, the effect of XIAP depletion in NCI-H460 cells ranged from approximately 20% [14] to 55% reduced viability [22]. The reported differences in phenotype upon XIAP knockdown for a given tumor cell line could be a function of degree and or duration of knockdown, the methodology for quantifying viability, or a more subtle parameter such as cell-culture conditions.

We present a systematic study of siRNA mediated knockdown of XIAP in human tumor cell lines of diverse tissue origin, including cell lines used in previous reports. In addition to assessing the effect of XIAP knockdown under normal growth conditions, we also explored whether loss of XIAP sensitizes tumor cells to either intrinsic or extrinsic inducers of cell death. Interestingly, loss of XIAP function sensitizes human tumor cell lines to TRAIL, but not inducers of the intrinsic death pathway.

## Methods

### Cell Lines

DU-145, HCT-116, MCF7, PC-3 and SW-620 were from the Division of Cancer Treatment and Diagnosis, National Cancer Institute (Frederick, Maryland). A-375, BxPC-3, LS 174T, and T24 were from American Type Culture Collection (Manassas, VA). PATU-I cells were from Dr. David Hockenberry at the Fred Hutchinson Cancer Research Center (Seattle, WA). All cell lines were maintained in RPMI 1640 medium (Invitrogen, Carlsbad, CA) supplemented with 10% fetal bovine serum (Invitrogen) at 37° with humidified air containing 5% CO_2_.

### siRNA Studies

**Figure 1:**
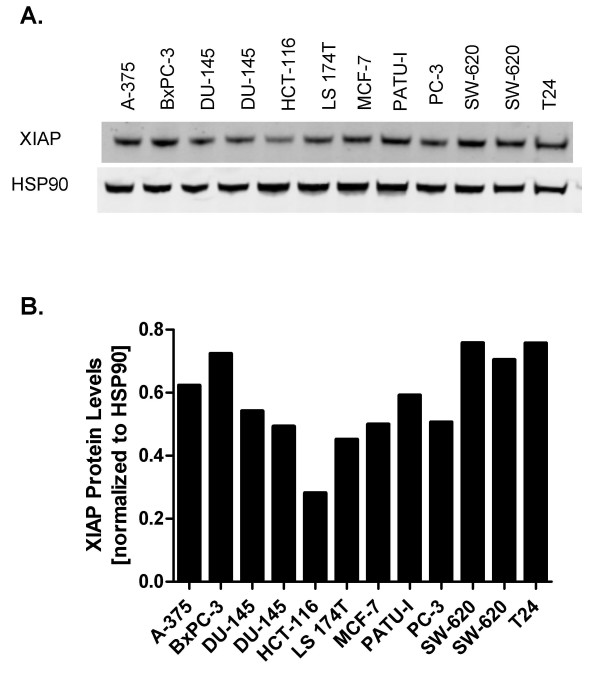
**XIAP protein levels in a panel of human tumor cell lines**. A. XIAP levels were monitored by Western blot from lysates of tumor cells. B. Quantification by LICOR Odyssey imaging. XIAP levels were normalized to the loading control HSP90 (Materials and Methods).

Transfections with siRNA: Silencer^® ^Select XIAP siRNAs were from Perkin Elmer Applied Biosystems (Foster City, CA). All XIAP depletions were carried out using Silencer^® ^Select siRNA ID# s1455, except in Figure 1 where siRNA ID# s1456 was compared to s1455. Silencer^® ^Negative Control #1 siRNA (Perkin Elmer,#AM4611) was included in each experiment. PLK1 siGENOME^® ^SMARTpool ^® ^siRNA (Cat. No. M-003290-01-0005) and Non-targeting pool (Cat. no D-001810-10-05) were from Thermo Scientific (Waltham, MA). Cells were trypsinized, washed in medium containing serum, adjusted to 5 × 10^6 ^cells/mL in RPMI 1640 supplemented with 10% fetal bovine serum. 2 × 10^6 ^cells in a 400 uL volume were transfected with siRNA by single pulse electroporation in a Gene Pulser^® ^Cuvette (Bio-Rad) with a 0.4 cm electrode gap using a Gene Pulser Xcell™ Electroporation system (Bio-Rad, Hercules, CA) set to 230 volts, 875 μF.

### Western Blot

Cells were lysed in modified radioimmunoprecipitation buffer (mRIPA; 10 mM Tris, 150 mM NaCl, 1% (v/v) NP-40, 0.5% deoxycholate, 0.1% SDS, 5 mM EDTA, pH 7.4) containing cOmplete protease inhibitor cocktail (Roche Applied Science, Indianapolis, IN) 48 hr after transfection. Total protein of the clarified lysates was quantitated by Lowry Assay (Biorad DC protein assay). NuPAGE^® ^LDS Sample Buffer and Sample Reducing Agent were added to the lysates and heated to 85°C for 10 mins. 20 μg of total cell protein was separated in NuPAGE 4-12% Bis Tris Gel and transferred to a nitrocellulose membrane using an iBlot^® ^Dry Blotting System (Invitrogen). Membranes were incubated in Blocking Buffer (LI-COR, Lincoln, NE) for 30 minutes at room temperature, followed by 60 minute incubations with mouse monoclonal antibodies against XIAP (BD Transduction Laboratories, Cat. No. 610716), Hsp90 (BD Transduction Laboratories, Cat. No. 610418), or PLK1 (Cell Signaling Technology Cat. No. 208G4). Following incubation with an IRDye^® ^Conjugated Goat Anti Mouse IgG (LI-COR), protein bands were quantified using an Odyssey^® ^Infrared Imaging System. Graphing of data to determine XIAP levels was performed using Graphpad PRISM^® ^software. Percent XIAP levels were calculated as follows:

% XIAP = (fluorescence XIAP band of sample-bkgrd/fluorescence Hsp90 band of sample-bkgrd) ÷ (fluorescence XIAP band of untreated cells-bkgrd/fluorescence Hsp90 of untreated cells-bkgrd)

### Viability Assays

After transfection, cells were immediately diluted in RPMI 1640 supplemented with 10% fetal bovine serum and added to clear bottom white wall 96 well plates at a concentration of 2500 cells/well in a 100 μL volume. 100 μL of ATPlite™ 1 step reagent was added to each well and the luminescence was measured using a Spectramax^®^L microplate luminometer. Data was acquired using SOFTMAX^®^Pro software. Graphing and statistical analysis was performed using Graphpad PRISM^® ^software. For experiments to determine TRAIL sensitivity, soluble human recombinant *Killer*TRAIL™ (Alexis Biochemicals, San Diego) was added at 32 hr after transfection; cells were incubated for an additional 24 hr prior to addition of ATPlite™.

## Results

### Efficient siRNA-mediated knockdown of XIAP protein levels

**Figure 2:**
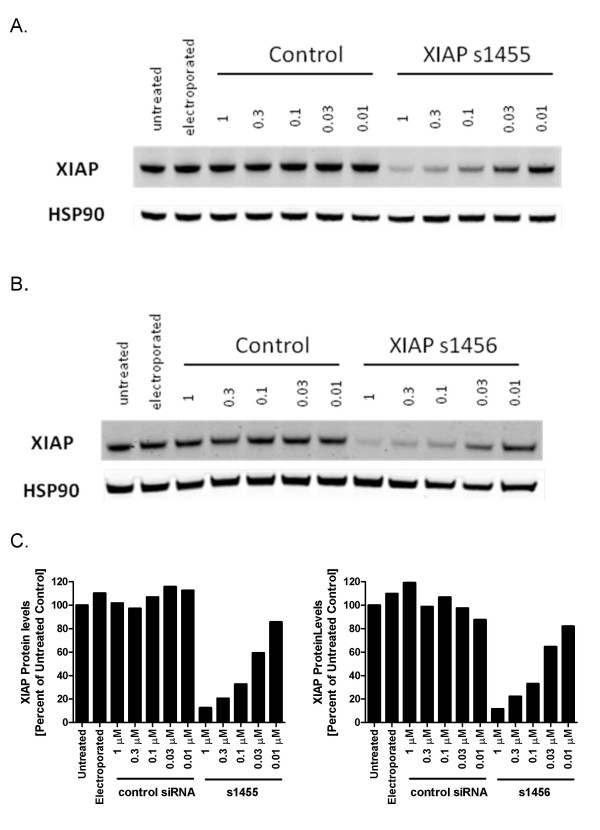
**Dose dependent increase in XIAP knockdown with XIAP siRNA s1455 and s1456**. SW620 cells were electroporated with the concentrations of siRNA indicated. After 48 hr, cells were lysed and XIAP levels were monitored by Western blot. A. s1455 B. s1456 C. Quantification by LICOR Odyssey imaging. Percent XIAP levels are expressed relative to the mean of untreated cells.

XIAP protein levels were monitored in 10 human tumor cell lines derived from different tumor types (Table 1 and Figure 1). The range of XIAP protein levels was relatively narrow, with only a 2.5-fold difference between the highest (e.g SW-620 and BxPC3) and lowest (e.g. HCT-116) expressing cell lines. The relatively high XIAP expression in SW-620 cells was consistent with published work, where it was also shown that these cells are resistant to TRAIL mediated apoptosis [23]. Moreover, reduction of XIAP protein levels by siRNA knockdown sensitized SW-620 cells to TRAIL killing. Based on these data, we chose SW-620 cells to optimize an electroporation-based siRNA protocol for efficient knockdown of XIAP protein. The cells were electroporated with increasing concentrations of the siRNAs s1455 and s1456 (Dharmacon Inc). Decreasing XIAP protein levels were correlated with increasing concentrations of both siRNAs (Figure 2). The dose-response for each siRNA was similar and the maximum knockdown was achieved at 1 μM; XIAP protein levels in s1455 and s1456 treated cells were 11.5% and 12.5% of untreated controls, respectively. Since there was no significant difference between the two siRNAs, we chose s1455 for all subsequent experiments, which were all performed with 1 μM of siRNA to ensure maximum knockdown. This concentration of siRNA is typical of studies that have employed electroporation [18,24,25]

**Table 1:** Human tumor cell lines.

Cell Line	Cancer Type	P53 Status
A-375^a^	Melanoma	functional

BxPC-3^b^	Pancreatic	mutant

PATU-1	Pancreatic	unknown

DU-145^c^	Prostate	mutant

PC-3^c^	Prostate	mutant

SW-620^c^	Colon	mutant

HCT-116^c^	Colon	functional

LS 174T^d^	Colon	functional

MCF7^c^	Breast	functional

T24^e^	Bladder	mutant

a [42], b [43], c [44], d [45], e [46]

**Figure 3:**
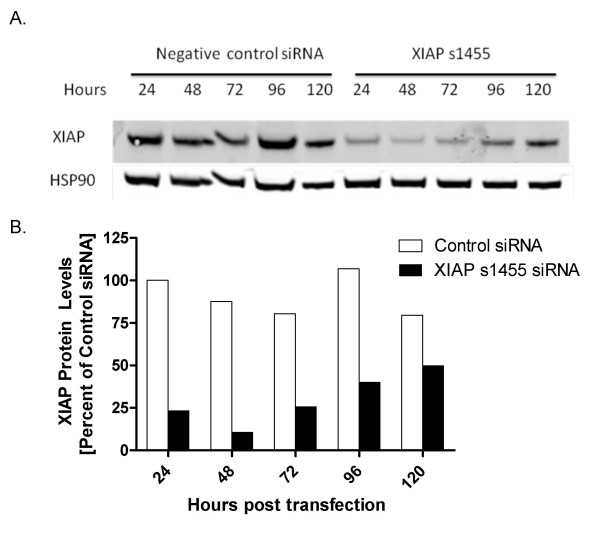
**Kinetics of siRNA mediated XIAP knockdown**. SW620 cells were electroporated with 1 μM s1455 or 1 μM control siRNA. Samples were collected every 24 hours and XIAP levels were detected by Western Blot (A) and (B) quantified by LICOR Odyssey imaging. XIAP values were normalized to the loading control, HSP90. Percent XIAP protein levels are expressed relative to the mean of control siRNA at 24 hr. 1 μM control siRNA (unfilled square) and 1 μM XIAP siRNA (black square).

The kinetics of XIAP knockdown was assessed over five days (Figure 3). A decrease in protein levels was evident at 24 hr, at 23% of untreated cells. A nadir of 10% was observed at 48 hr. Recovery began by 72 hr, with XIAP levels of 26%, 40%, and 50% of controls detected at 72, 96, and 120 hr, respectively.

### Effect of XIAP protein knockdown on tumor cell viability

**Figure 4:**
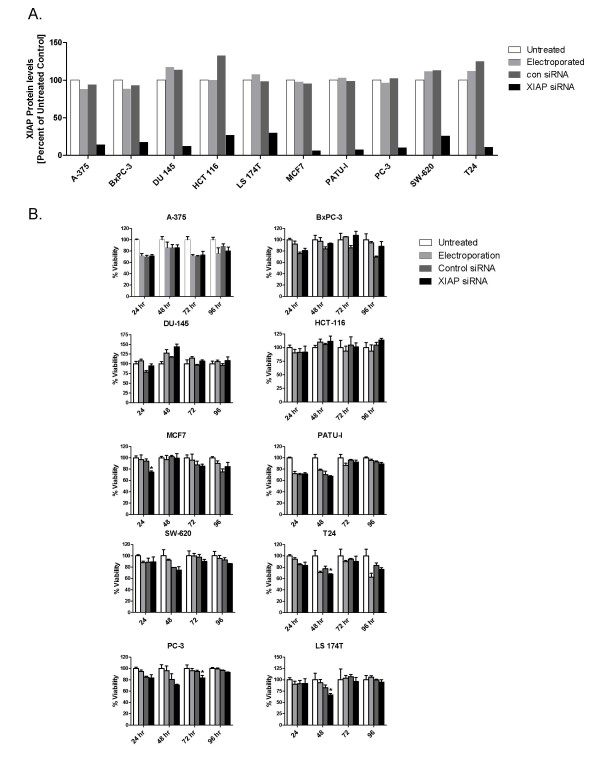
**XIAP depletion in a panel of tumor cell lines**. A. XIAP levels were monitored by Western blot from lysates of untreated cells (unfilled square), electroporated cells, (grey square), cells electroporated with 1 μM control siRNA (dark grey square), or 1 μM s1455 XIAP siRNA (black square) 48 hr following electroporation. XIAP levels were quantified by LICOR Odyssey imaging. Percent XIAP levels are expressed relative to the untreated control for each cell line. B. Viablity of XIAP depleted cells was measured at 24, 48, 72 and 96 hr post electroporation; untreated cells (unfilled square), electroporated cells (light grey square), cells electroporated with 1 μM control siRNA (dark grey square), or 1 μM s1455 XIAP siRNA (black square).

The effect of XIAP depletion on viability of the 10 human tumor cell lines was measured at various time points over 96 hr. These cell lines were either wild-type or mutant at the tumor suppressor gene, p53 (Table 1). Some reports have indicated that wild-type p53 status correlates with sensitivity to XIAP knockdown [26,27]. Cells were electroporated with 1 μM XIAP siRNA s1455 or control siRNA. XIAP levels in cells treated with s1455 siRNA ranged from 6% to 26% of untreated cells at 48 hr (Figure 4A). In 6 of the 10 cell lines, there was no significant difference in viability at any time point between the XIAP depleted cells and control siRNA treated cells (Figure 4B). In the remaining 4 cell lines there was a modest, but statistically significant, 10-20% decrease in cell viability, albeit at only one of the time points. In MCF-7 cells there was a 20% decrease in viability at 24 hr and in PC-3 cells a 12% decrease at 72 hr. At all other time points, cell number in s1455 treated MCF-7 and PC-3 cells was equivalent to that of control siRNA treated cells. Similarly, in both LS-174T and T24 cells there was a 20% and 13% decrease in viability, at 48 hr, respectively, that was not observed at the other time points. As a positive control for siRNA mediated tumor cell death, SW620 cells were electroporated with a PLK1 siRNA (1 μM). As expected for this well validated cancer target [28,29] there was a 64%, 75% and 84% decrease in viability at 48, 72 and 96 hr post electroporation, respectively. (75% PLK1 knockdown at 48 hr; Additional File 1). The lack of effect in the majority of the cell lines, and the modest and transient nature of the decreased viability in the other cell lines, suggests that in many tumor cell lines under normal in vitro growth conditions, XIAP has no essential role regulating proliferation or survival.

### XIAP depleted cells are sensitized to TRAIL but not intrinsic pathway inducing agents

**Figure 5:**
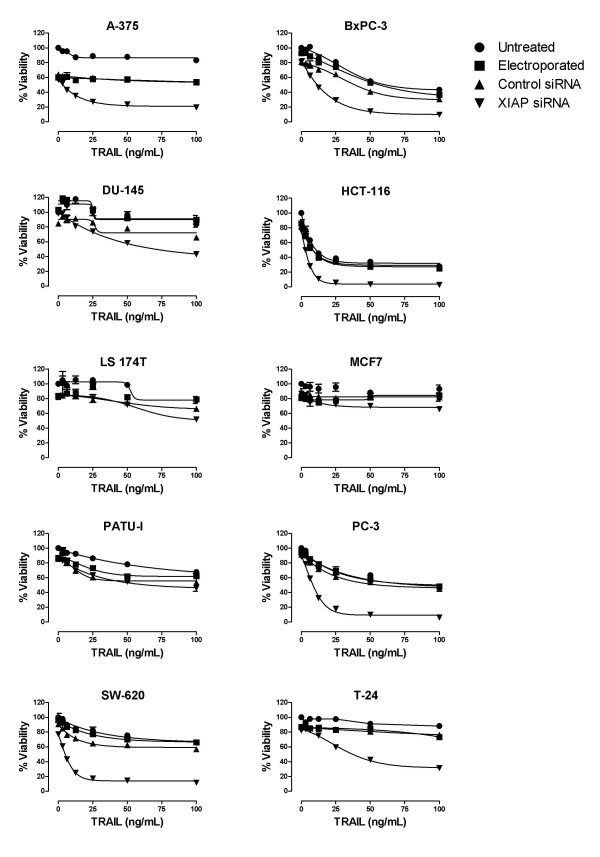
**Effect of XIAP depletion on tumor cell viability and TRAIL sensitivity**. XIAP depleted cells were exposed to TRAIL 40 hr following electroporation, and viability was measured 24 hr following TRAIL exposure Untreated cells (black circle); Electroporated cells (black square); 1 μM control siRNA (black triangle) 1 μM XIAP siRNA (inverted black triangle). Percent viability is expressed relative to untreated controls for each cell line. A similar result was obtained with the s1456 siRNA in SW620 cells (Additional File 2).

Several studies have reported XIAP depletion increases sensitivity to TRAIL mediated apoptosis [10,14,17,23,30-32]. We exposed s1455 treated cells to TRAIL to determine if XIAP knockdown was sufficient to sensitize them (Figure 5). In 6 of 10 cell lines, XIAP depletion increased sensitivity to TRAIL mediated death, indicating that the death receptor pathway is functional in those cells and that XIAP functions as a negative regulator of caspase-8 mediated cell death. Similar results were obtained in the SW-620 cell line with the s1456 siRNA indicating that the observed effects were not specific to siRNA s1455 (Additional File 2). Minimal or no sensitivity to TRAIL was observed in the other 4 cell lines, with or without XIAP knockdown. The lack of TRAIL mediated killing in these other cell lines may result from several possibilities, such as insufficient death receptor expression (DR4 and DR5), the glycosylaton state of these receptors or high decoy receptor expression [33,34]. In the one resistant line (DU-145) for which there is publicly available gene expression data http://dtp.nci.nih.gov/mtweb/search.jsp, the DR4/DR5 expression is similar to the other cell lines that were also sourced from the NCI-60 panel (e.g. SW-620) and thus would appear to be sufficient to engage TRAIL mediated killing. To determine whether the degree to which XIAP knockdown sensitized cells to TRAIL was additive or synergistic in nature, we determined the viability of SW620 cells with a titration of both s1455 XIAP siRNA and TRAIL alone and in combination (Additional File 3). The Combination Index [35] indicated synergy at all 3 titrations.

**Figure 6:**
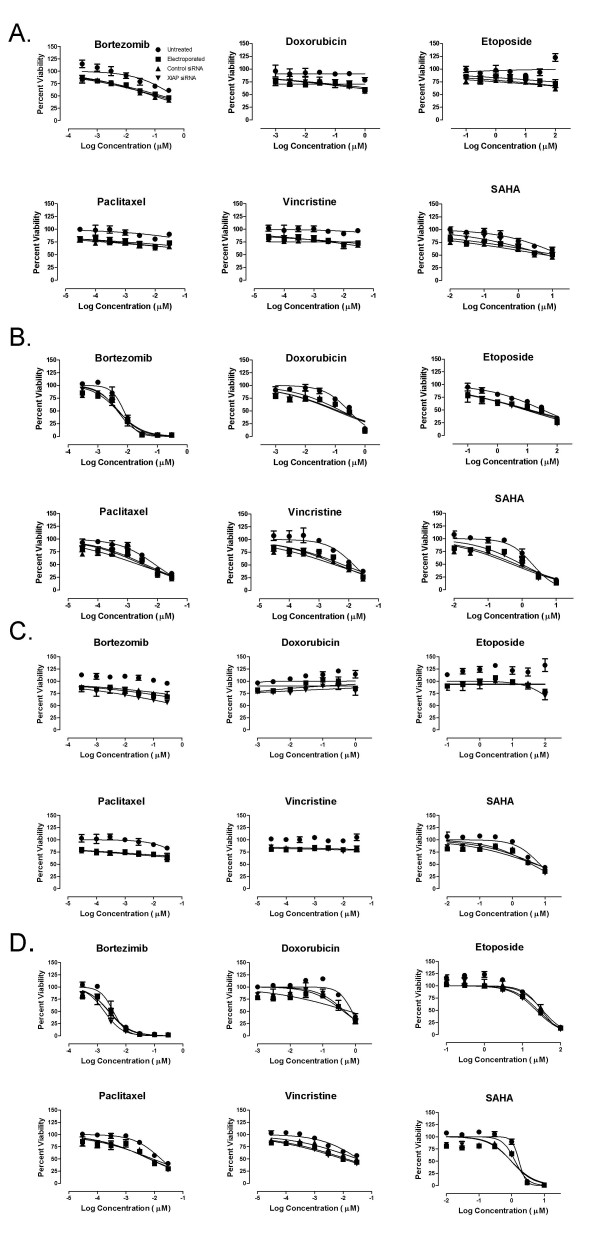
**Effect of XIAP depletion on chemosensitivity of HCT-116 and SW620 cells**. Cells were electroporated with 1 μM control siRNA (con siRNA) or 1 μM s1455 XIAP siRNA. HCT-116 and SW-620 were treated with an array of chemotherapeutic agents for 24 hr (A and C, respectively) or 48 hr (B and D). For the 24 hr exposure, the agents were added 32 hr following electroporation. For the 48 hr exposure, the agents were added 24 hr following electroporation. Untreated cells (black circle); Electroporated cells (black square); 1 μM control siRNA (black triangle) XIAP siRNA (inverted black triangle).

To explore the role of XIAP as a negative regulator of apoptosis mediated through the intrinsic pathway, we treated XIAP-depleted HCT-116 and SW-620 cells with a variety of standard-of-care chemotherapeutics, the proteasome inhibitor bortezomib and the HDAC inhibitor SAHA. All of these agents are thought to engage the mitochondrial-based, intrinsic pathway, although by distinct mechanisms [36-39]. Twenty four hours post electroporation, cells were treated with varying concentrations of the therapeutic compounds. Cell viability was determined at both 24 and 48 hours post compound addition, thus ensuring that cells were exposed to the agents during maximal XIAP knockdown. None of the compounds significantly impacted cell viability in either cell line at 24 hr (Figure 6A and 6C). By contrast, significant dose-dependent cytotoxicity was observed at 48 hr for all agents (Figure 6B and 6D). In contrast to the combined effect of XIAP knockdown and TRAIL, no significant increase in cytotoxicity was observed when these agents were combined with XIAP knockdown, compared with the various control groups (Figure 6B and 6D; Table 2 Similar results were obtained with these same agents in the PC-3 prostate cancer cell line (Additional File 4) Table 2 To verify efficient knockdown in the HCT-116 and SW-620 cells, XIAP protein levels were determined in parallel cultures of both cell lines at 48 hr post electroporation (knockdown nadir). XIAP mRNA was efficiently targeted in these cells with XIAP protein levels at 13% and 17% of the untreated SW-620 and HCT-116 cells, respectively (Additional File 5).

**Table 2:** Sensitivity of XIAP depleted HCT-116, SW-620 and PC-3 tumor cells to various mechanistically distinct anti-cancer agents.

Drug	Cell line	EC_50 _(μM)			
		Untreated	Electroporated	Control siRNA	XIAP siRNA
Bortezomib	HCT-116	0.0075	0.0050	0.0047	0.0045

Doxorubicin	HCT-116	0.29	0.13	0.16	0.11

Etoposide	HCT-116	34.11	12.99	10.62	8.90

Paclitaxel	HCT-116	0.0083	0.0040	0.0024	0.0031

SAHA	HCT-116	2.014	0.98	0.67	0.51

Vincristine	HCT-116	0.015	0.0067	0.0035	0.0045

Bortezomib	SW-620	0.0036	0.0025	0.0025	0.0017

Doxorubicin	SW-620	0.79	0.657	0.48	0.43

Etoposide	SW-620	31.79	25.55	33.68	22.70

Paclitaxel	SW-620	0.017	0.0067	0.0086	0.0064

SAHA	SW-620	1.69	0.98	1.29	0.97

Vincristine	SW-620	0.035	0.013	0.031	0.0095

Bortezomib	PC-3	0.01	0.0063	0.0037	0.0041

Doxorubicin	PC-3	0.15	0.073	0.038	0.038

Etoposide	PC-3	41.96	16.58	26.13	16.76

Paclitaxel	PC-3	0.0028	0.00090	0.00067	0.00037

SAHA	PC-3	1.762	0.72	0.068	0.53

Vincristine	PC-3	0.0025	0.0016	0.0016	0.0016

Potency (EC_50_) determinations from the dose response curves in Figure 5 (HCT-116 and SW-620) and Additional File 5 (PC3) were determined using non-linear regression and a least squares fit (GraphPad Prism software)

## Discussion

Here we report that transient, siRNA-mediated depletion of XIAP alone does not significantly decrease human tumor cell viability. We interpret the results to mean that XIAP does not have an essential role in growth and survival of tumor cell lines under normal, optimized growth conditions *in vitro*. This conclusion is consistent with a lack of effect on developmental apoptosis in mice harboring a germ line XIAP mutation and in transformed mouse embryo fibroblasts derived from these XIAP knockout mice [9]. Similar results were obtained with human colorectal cancer cells in which the XIAP locus was deleted via homologous recombination [10]. However, in these studies and others using transient or stable XIAP knockdown, loss of XIAP function sensitized the cells to TRAIL induced apoptosis. Our study is a more expansive survey, and supports the idea that XIAP has a critical role in negatively regulating death receptor mediated apoptosis across a wide array of tumor cell lines derived from diverse tissue types. Surprisingly, similar enhancement of apoptosis was not observed with multiple mechanistically distinct chemotherapeutics or the proteasome inhibitor bortezomib, or the HDAC inhibitor SAHA. All of these agents are thought to induce apoptosis predominantly (but perhaps not exclusively) through the mitochondrial pathway, involving cytochrome c and SMAC release and subsequent activation of caspase-9 by the apoptosome. Our data strongly suggest that XIAP has a more central role in inhibiting the extrinsic caspase-8 mediated death pathway than the intrinsic, caspase-9 dependent pathway. One potential explanation for the difference between extrinsic versus intrinsic death inducers is that the latter cause a release of SMAC, an endogenous inhibitor of XIAP. In wild-type cells, the caspase inhibitory activity of XIAP may be neutralized by SMAC following a robust intrinsic death pathway signal, essentially mimicking XIAP depletion. Therefore, no further increase in apoptotic response would be expected in XIAP siRNA treated cells. Support of this hypothesis comes from the elegant studies of the Prehn group, where loss of XIAP function in staurosporine treated HeLa cells did not accelerate substrate cleavage after detection of mitochondrial outer membrane permeabilization [40].

In contrast to our studies, Ras/E1A transformed MEFs derived from XIAP KO mice exhibited an increased sensitivity to the apoptosis inducing effects of etoposide compared to their wild-type counterparts [9]. It is possible that Ras/E1A transformed MEFs are under different apoptotic pressures than the human cancer cells used in our study, resulting in XIAP having a more central role in suppressing intrinsic pathway mediated cell death. Testing the effects of other mechanistically distinct inducers of the intrinsic cell death pathway in Ras/E1A transformed MEFs should help clarify this and determine if the observed effects in MEFs are specific to etoposide.

Yang et al [8] reported that several cell lines, including a subset of those used in this study (BxPC-3, MCF-7, and SW-620) exhibited high basal levels of activated caspase-3 and -8 activity in the absence of other markers of apoptosis. It was argued that these cells were non-apoptotic via a compensatory increase in XIAP expression, which neutralized the caspase activity. Within the same study, over-expression of XIAP-associated factor 1 (XAF-1) in MCF-10A and MDA-MB-231 resulted in an increase in apoptosis. However, the biological activities of XAF-1 are complex and not yet fully elucidated, and thus it is difficult to ascertain whether this increase in cell death is solely mediated by XIAP. The more definitive XIAP knockdown experiments were not performed. If viable tumor cells such as BxPC3 and SW620 do in fact have activated caspases, our data suggests that these "death enzymes" are unlikely to be directly inhibited by XIAP, but rather by some other mechanism. Alternatively, in the context of XIAP knockdown the level of active caspases is still below a threshold necessary to induce cell death. Since 100% knockdown is never achieved with siRNA, the residual XIAP protein in the siRNA treated cells may be sufficient to inhibit the activated caspases present in these cells.

Several authors have reported that functional p53 is required for XIAP depletion to result in cell death. Tong and colleagues [26] found that the p53 positive MKN-45 gastric carcinoma cell line exhibited an elevated apoptotic rate following XIAP depletion, while the p53 mutant cell line MKN-28 was unaffected. Mohapatra and colleagues [27] reported that XIAP depletion did not result in increased apoptosis in p53 wild type LNCaP or p53 deficient PC-3 prostate cancer cells although over-expression of p53 in both cell lines resulted in apoptosis following XIAP depletion. Our studies included cell lines that harbor wild-type and mutant (loss-of-function) p53, however, there was no obvious correlation between response to XIAP knockdown and p53 status.

Recently, multiple reports indicated that tumor cell death induced by multiple, chemically distinct SMAC mimetics was in fact dependent on the proteasomal degradation of multiple members of the IAP family and subsequent induction of TNFα production and caspase-8 mediated death [41]. Treated cells that did not have detectable levels of TNFα did not undergo apoptosis nor did TNFα-positive cells that were simultaneously treated with a TNFα blocking antibody. These results lend support to our conclusions from the knockdown experiments that under normal growth conditions in vitro, most tumor cells have not sufficiently engaged an apoptotic pathway such that their survival is dependent on XIAP. Some other death signal is needed (e.g. TNFα production or exogenous TRAIL), which, together with XIAP antagonism results in enhanced apoptosis. One outstanding question is whether the anti-tumor activity of the SMAC mimetics *in vivo *is also dependent on engagement of the TNFα pathway. It is possible that the associated stresses of *in vivo *tumor growth (e.g. hypoxia) generate a death signal (activated caspases) that is sufficient to render the tumor cells sensitive to inhibition of XIAP solely via the disruption of the caspase 9/XIAP interaction. In support of this notion, multiple reports have shown that stable shRNA or antisense knockdown of XIAP resulted in decreased tumor cell growth, as subcutaneous xenografts *in vivo*, but not as culture mono-layers, *in vitro *[14,18,32]. *In vivo *studies with inducible shRNAs that target XIAP in both nascent and established tumors may help resolve this issue, and should provide further insight for validation of XIAP as a cancer drug target.

## Conclusion

Our work is consistent with others and predicts that agents that simply disrupt the caspase-3/9-XIAP interaction may hold limited therapeutic promise as monotherapy and that their utility will be likely found in the combination setting, in particular with therapies that engage the extrinsic death receptor pathway. Ultimate validation of XIAP as a cancer drug target will come from the clinical development of both the SMAC mimetics and the anti-sense based XIAP cancer therapies, both of which have recently entered Phase I clinical trials.

## Competing interests

FLS and RP are employees and shareholders of Receptos (formerly Apoptos). JS and JHH have no competing interests.

## Authors' contributions

JS designed and carried out experiments, assembled figures and wrote the paper. FLS contributed with experimental design, analyzed data and helped edit the paper. RP contributed with experimental design and helped edit the paper. JHH contributed with experimental design, analyzed data and wrote the paper. All authors have read and approved the final manuscript.

## Pre-publication history

The pre-publication history for this paper can be accessed here:

http://www.biomedcentral.com/1471-2407/10/11/prepub

## Supplementary Material

Additional file 1 **Effect of depletion of Polo-like kinase 1 (PLK1) on cell viability**. SW-620 cells were electroporated with the concentrations of siRNA indicated. (A and B). After 48 hr, cells were lysed, and PLK1 protein levels were detected by Western blot and quantified by LICOR Odyssey imaging. C. Cell viability was measured at 24 hr (unfilled square), 48 hr (light grey square), 72 hr (dark grey square) and 96 hr (black square). Click here for file

Additional file 2 **Effect of XIAP depletion on viability and TRAIL sensitivity in SW620 cells using an alternate siRNA**. A. Lysates of SW620 and HCT-116 cells were collected 48 hr following electroporation with 1 μM control siRNA or XIAP siRNA s1456, and XIAP levels detected by western blot and quantified using LICOR Odyssey imaging (B). Untreated cells (unfilled square), electroporated cells (light grey square), cells electroporated with 1 μM control siRNA (dark grey square), or 1 μM s1456 XIAP siRNA (black square). Percent XIAP levels are expressed relative to the untreated control. C. Viability of XIAP depleted cells was measured at 24, 48, 72 and 96 hr. Untreated cells (unfilled square), electroporated cells (light grey square), cells electroporated with 1 μM control siRNA (dark grey square), or 1 μM s1456 XIAP siRNA (black square). D. XIAP depleted cells were exposed to TRAIL 40 hours following electroporation, and viability was measured after 16 hr. Percent viability is expressed relative to untreated controls. Untreated cells (black circle), electroporated cells (black square), cells electroporated with 1 μM control siRNA (black triangle), or 1 μM s1456 XIAP siRNA (inverted black triangle). Click here for file

Additional file 3 **Measurement of synergism of XIAP depletion and TRAIL on viability of SW620 cells**. Cells were electroporated with varying concentrations of siRNA and TRAIL was added 40 hr post electroporation. A and B. Viability was measured using ATPlite 16 hr following addition of TRAIL. Combination index was determined using Compusyn software. A combination index of < 0.1 is indicative of very strong synergism. (Chou et. al., 2006). 1 μM s1456 XIAP siRNA (black circle). TRAIL (black square) TRAIL and 1 μM s1456 XIAP siRNA (black triangle). C. XIAP protein levels at 48 hr post electroporation. Click here for file

Additional file 4 **Effect of XIAP depletion on chemosensitivity of PC-3 cells**. Cells were electroporated with 1 μM control siRNA (con siRNA) or 1 μM s1455 XIAP siRNA. All compounds were added 24 hr following electroporation and incubated for 72 hr. Untreated cells (black circle); Electroporated cells (black square); 1 μM control siRNA (black triangle) XIAP siRNA (inverted black triangle). Click here for file

Additional file 5 **XIAP protein levels in XIAP depleted SW620 and HCT-116 cells**. Lysates of SW620 and HCT-116 cells were collected 48 hours following electroporation with 1 μM control siRNA or XIAP siRNA s1455, and XIAP protein levels detected by Western blot (A) and quantified using LICOR Odyssey imaging (B). Parallel cultures from the same electroporations were used to determine XIAP depletion on chemosensitivity (Figure 5). Percent XIAP protein levels are expressed relative to the mean of untreated controls. Click here for file
