# Supplementary figures and images for: XIAP is not required for human tumor cell survival in the absence of an exogenous death signal
Source: BMC Cancer. 2010 Jan 12;10:11. doi: 10.1186/1471-2407-10-11 (PMC2827368; doi:10.1186/1471-2407-10-11)

## Slide 1
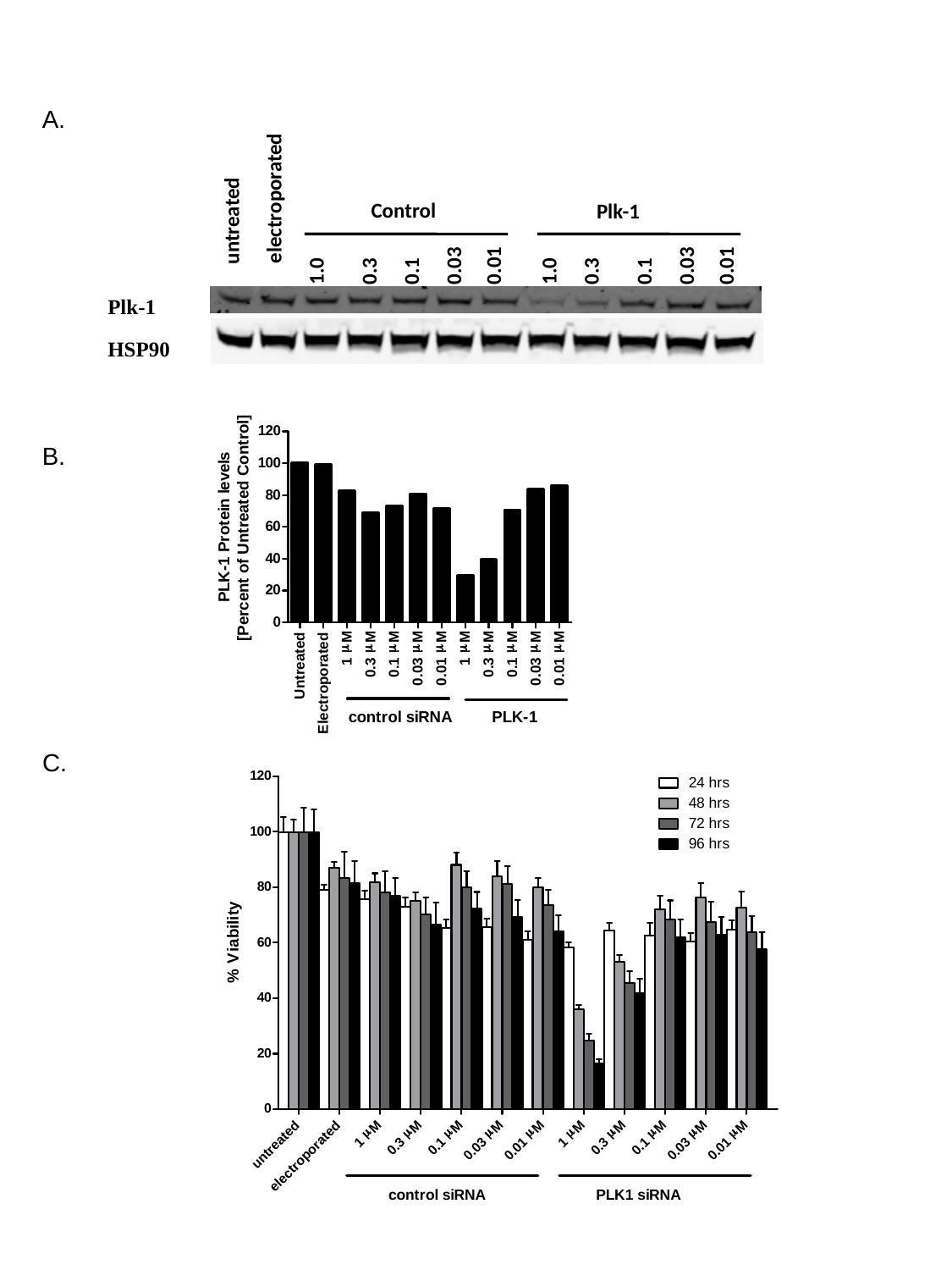

A.
electroporated
Control
Plk-1
 untreated
0.03
0.03
0.01
0.01
0.3
0.3
0.1
0.1
1.0
1.0
Plk-1
 HSP90
B.
C.

Supplement: Additional file 1 — Effect of depletion of Polo-like kinase 1 (PLK1) on cell viability. SW-620 cells were electroporated with the concentrations of siRNA indicated. (A and B). After 48 hr, cells were lysed, and PLK1 protein levels were detected by Western blot and quantified by LICOR Odyssey imaging. C. Cell viability was measured at 24 hr (unfilled square), 48 hr (light grey square), 72 hr (dark grey square) and 96 hr (black square). [file 1471-2407-10-11-S1.PPT]

## Slide 1
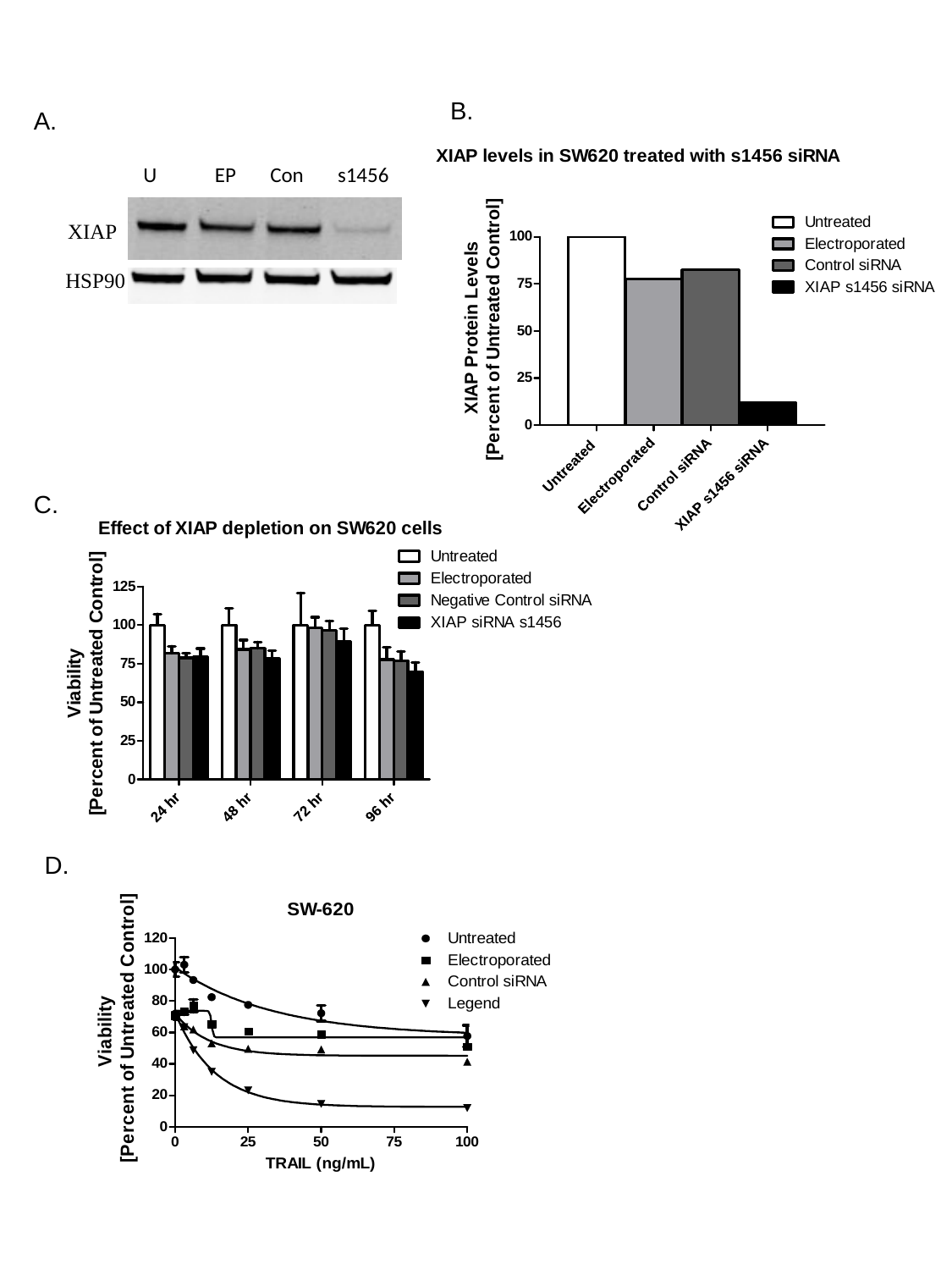

B.
A.
 U EP Con s1456
XIAP
 HSP90
C.
D.

Supplement: Additional file 2 — Effect of XIAP depletion on viability and TRAIL sensitivity in SW620 cells using an alternate siRNA. A. Lysates of SW620 and HCT-116 cells were collected 48 hr following electroporation with 1 μM control siRNA or XIAP siRNA s1456, and XIAP levels detected by western blot and quantified using LICOR Odyssey imaging (B). Untreated cells (unfilled square), electroporated cells (light grey square), cells electroporated with 1 μM control siRNA (dark grey square), or 1 μM s1456 XIAP siRNA (black square). Percent XIAP levels are expressed relative to the untreated control. C. Viability of XIAP depleted cells was measured at 24, 48, 72 and 96 hr. Untreated cells (unfilled square), electroporated cells (light grey square), cells electroporated with 1 μM control siRNA (dark grey square), or 1 μM s1456 XIAP siRNA (black square). D. XIAP depleted cells were exposed to TRAIL 40 hours following electroporation, and viability was measured after 16 hr. Percent viability is expressed relative to untreated controls. Untreated cells (black circle), electroporated cells (black square), cells electroporated with 1 μM control siRNA (black triangle), or 1 μM s1456 XIAP siRNA (inverted black triangle). [file 1471-2407-10-11-S2.PPT]

## Slide 1
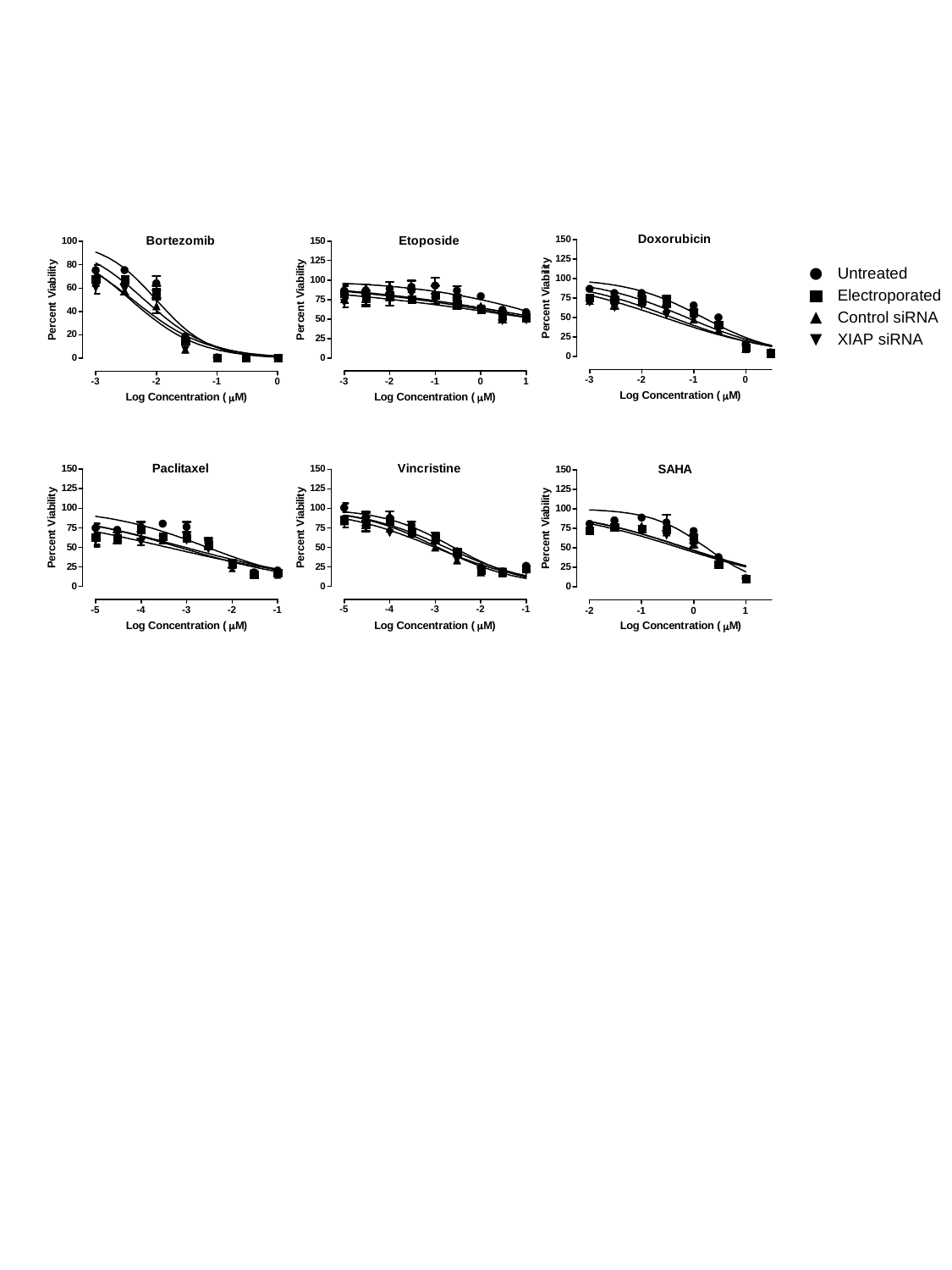

Supplement: Additional file 4 — Effect of XIAP depletion on chemosensitivity of PC-3 cells. Cells were electroporated with 1 μM control siRNA (con siRNA) or 1 μM s1455 XIAP siRNA. All compounds were added 24 hr following electroporation and incubated for 72 hr. Untreated cells (black circle); Electroporated cells (black square); 1 μM control siRNA (black triangle) XIAP siRNA (inverted black triangle). [file 1471-2407-10-11-S4.PPT]

## Slide 1
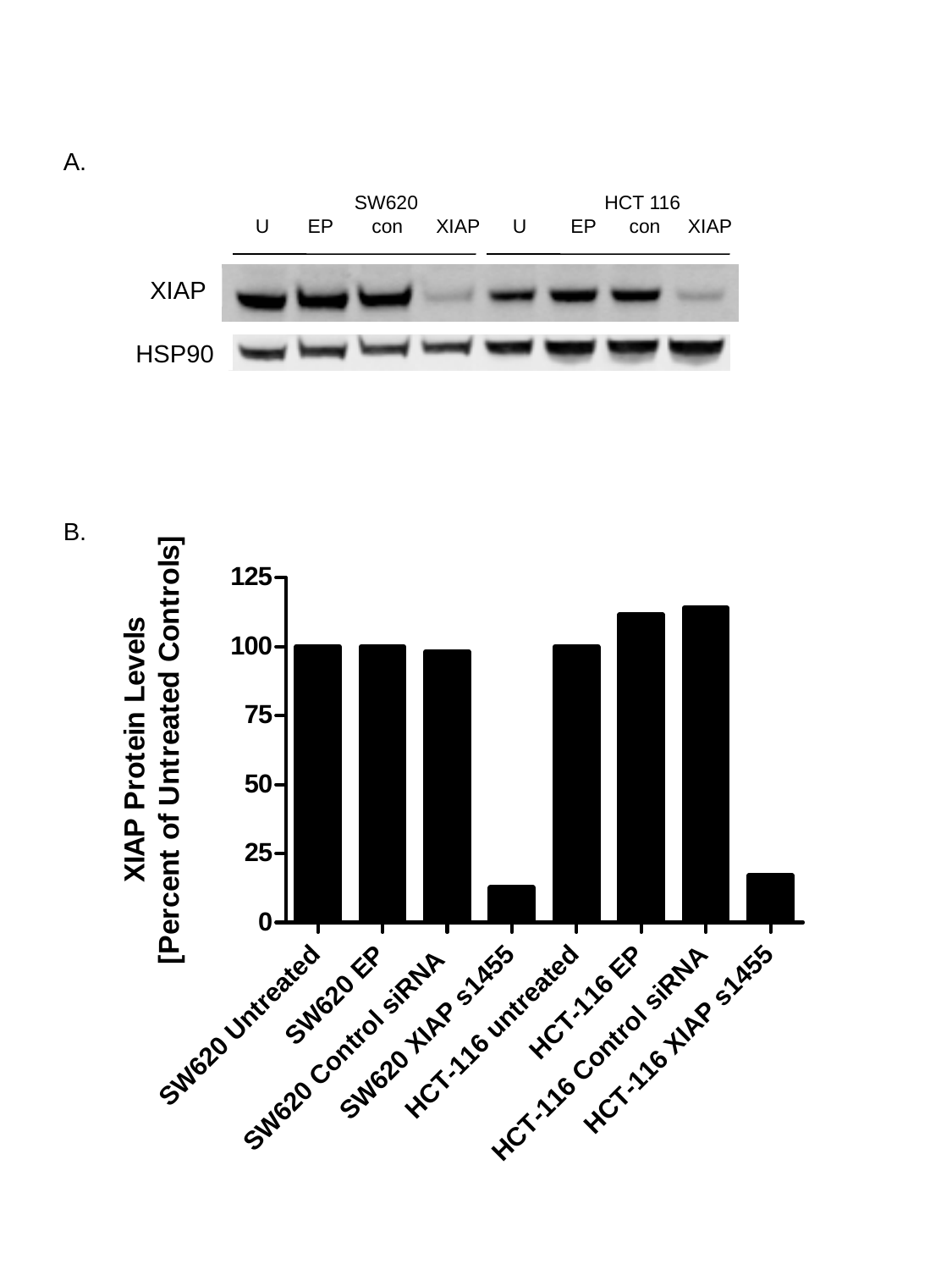

A.
 SW620 HCT 116
U EP con XIAP U EP con XIAP
XIAP
 HSP90
B.

Supplement: Additional file 5 — XIAP protein levels in XIAP depleted SW620 and HCT-116 cells. Lysates of SW620 and HCT-116 cells were collected 48 hours following electroporation with 1 μM control siRNA or XIAP siRNA s1455, and XIAP protein levels detected by Western blot (A) and quantified using LICOR Odyssey imaging (B). Parallel cultures from the same electroporations were used to determine XIAP depletion on chemosensitivity (Figure 5). Percent XIAP protein levels are expressed relative to the mean of untreated controls. [file 1471-2407-10-11-S5.PPT]
