# Supplementary material for: XIAP is not required for human tumor cell survival in the absence of an exogenous death signal
Source: BMC Cancer. 2010 Jan 12;10:11. doi: 10.1186/1471-2407-10-11 (PMC2827368; doi:10.1186/1471-2407-10-11)
Supplement: Additional file 3 — Measurement of synergism of XIAP depletion and TRAIL on viability of SW620 cells. Cells were electroporated with varying concentrations of siRNA and TRAIL was added 40 hr post electroporation. A and B. Viability was measured using ATPlite 16 hr following addition of TRAIL. Combination index was determined using Compusyn software. A combination index of < 0.1 is indicative of very strong synergism. (Chou et. al., 2006). 1 μM s1456 XIAP siRNA (black circle). TRAIL (black square) TRAIL and 1 μM s1456 XIAP siRNA (black triangle). C. XIAP protein levels at 48 hr post electroporation. [file 1471-2407-10-11-S3.PPT]

## Slide 1
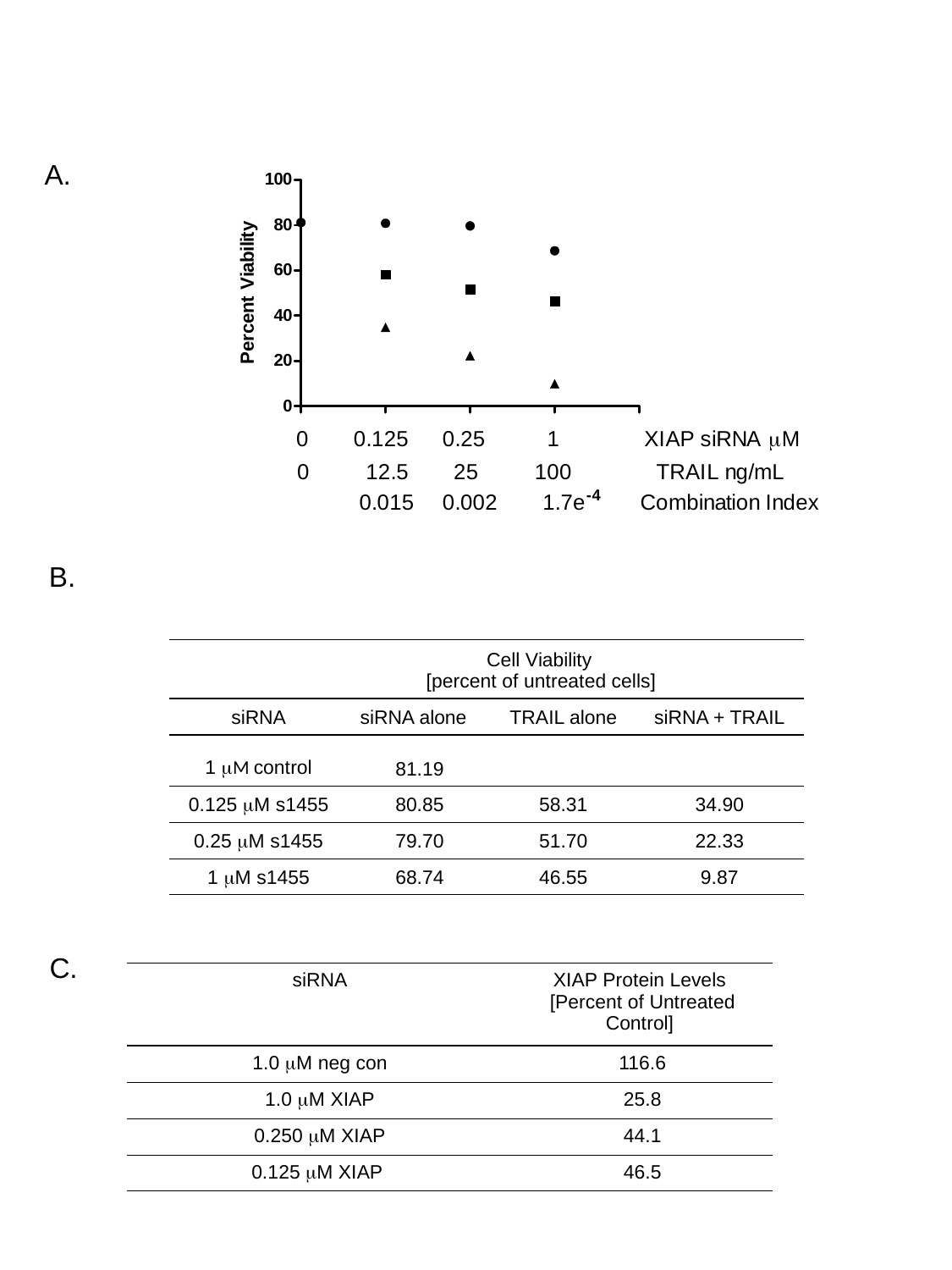

A.
B.
| | Cell Viability [percent of untreated cells] | | |
| --- | --- | --- | --- |
| siRNA | siRNA alone | TRAIL alone | siRNA + TRAIL |
| 1 μM control | 81.19 | | |
| 0.125 μM s1455 | 80.85 | 58.31 | 34.90 |
| 0.25 μM s1455 | 79.70 | 51.70 | 22.33 |
| 1 μM s1455 | 68.74 | 46.55 | 9.87 |
C.
| siRNA | XIAP Protein Levels [Percent of Untreated Control] |
| --- | --- |
| 1.0 μM neg con | 116.6 |
| 1.0 μM XIAP | 25.8 |
| 0.250 μM XIAP | 44.1 |
| 0.125 μM XIAP | 46.5 |
